# A novel Wnt/β-catenin signaling gene signature for progression and metastasis of gastric cancer

**DOI:** 10.32604/or.2024.054366

**Published:** 2025-04-18

**Authors:** JIA CHEN, FEI JIANG, KAIYI NIU, HAODONG ZHAO, LI LI, HONGZHU YU

**Affiliations:** 1 Department of General Surgery, Fuyang Hospital Affiliated of Anhui Medical University, Fuyang, 236000, China; 2 Department of Emergency Surgery, The First Affiliated Hospital of Anhui Medical University, Hefei, 230032, China; 3 Hepatopancreatobiliary Center, The Second Affiliated Hospital of Nanjing Medical University, Nanjing, 210000, China

**Keywords:** Gastric Cancer (GC), Signature genes, Prognosis, Carboxypeptidase Z (CPZ), Adipocyte Enhancer Binding Protein 1(AEBP1)

## Abstract

**Backgrounds:**

As cancer progresses through various stages of malignancy, metastasis, and drug resistance, the Wnt/-catenin signaling is frequently dysregulated. Despite advancements in medical technology and therapeutic strategies, the prognosis for numerous gastric cancer patients remains unfavorable.

**Methods:**

For the analysis of prognostic signature genes associated with Wnt signaling in GC, we used LASSO (least absolute shrinkage and selection operator) regression. To explore the function, cell specificity, and transcriptional regulation of the signature gene Carboxypeptidase Z (CPZ), we conducted co-expression analysis, single-cell RNA sequencing data analysis, transcription factor prediction, and dual luciferase reporter assay. The knockdown and overexpression experiments were also performed to observe the changes in the downstream gene expression, as well as the influence on the biological functions of GC cells.

**Results:**

We identified a five-gene signature, including CPZ, Collagen Triple Helix Repeat Containing-1 (CTHRC1), Dickkopf-1 (DKK1), Epidermal Growth Factor (EGF), and Glypican Proteoglycan-3 (GPC3), with risk scores predictive of the prognosis of GC patients. We found that the adipocyte enhancer binding protein 1 (AEBP1) and transcription factor 3 (TCF3) could interact in the nucleus and synergistically enhance the expression of Wnt signaling-associated genes, including WNT2/FZD2 (Wnt family member 2/frizzled class receptor 2) and VIM (vimentin), thus promoting the invasion, migration, and malignant metastasis of GC.

**Conclusions:**

Our study offers a precise gene-signature prediction method for the prognosis of GC. We discovered the synergistic effect of AEBP1 and TCF3 in the nucleus on GC metastasis. GC may benefit from the identification of this potential therapeutic target.

## Introduction

Although gastric cancer (GC) has shown a decline in fatality and occurrence rates over the past few decades, it remains the fifth most common malignancy and the fourth leading cancer killer worldwide, which has become a serious public health burden [1,2]. The lack of typical clinical manifestations of GC in the initial stage leads to delays in diagnosis and treatment as well as poor prognosis [3]. Although great efforts have been made in the prevention and treatment strategies of GC, including improved dietary habits, early detection, and effective treatment strategies [4], the prognosis for GC patients remains generally poor. Therefore, a deep understanding of the pathogenesis and discovery of new prognostic genes is necessary. With the advent of whole-genome sequencing and other high-throughput technologies, researchers have identified numerous genes associated with prognosis in specific cancers, providing clinicians with refined tools for predicting patient survival and treatment response [5].

Since the discovery of the first Wnt family member in 1982, Wnt signaling, essential for embryonic development, tissue repair, and cancer formation, has consistently been a focal point of research [6]. By activating the canonical Wnt pathway, β-catenin is stabilized and translocated to the nucleus, thus promoting the expression of genes involved in cell proliferation, differentiation, and migration [7]. A variety of tumors can be initiated and progressed by aberrant activation of Wnt signaling. For instance, studies show that PAPSS2 (3′-Phosphoadenosine 5′-phosphosulfate (PAPS) synthase 2) plays a protective role in intestinal carcinogenesis by antagonizing Wnt/β-catenin signaling [8]. Besides, α-Ketoglutarate (aKG) can promote hypomethylation of histone H3K4me3, leading to down-regulation of Wnt target genes, which inhibits Wnt signaling and significantly restricts colorectal cancer (CRC) growth [9]. The Wnt-β-catenin signaling could also control ferroptosis to facilitate GC chemotherapy resistance [10]. Although many achievements have been obtained in the field of Wnt signaling and cancers, further investigation of Wnt signaling in GC is significant to explore more effective treatment and prognosis prediction strategies.

The Adipocyte Enhancer Binding Protein 1 (AEBP1) is a multifunctional protein involved in a variety of cellular processes [11–15], including adipogenesis, inflammation, and vascular remodeling. Previous studies indicate that AEBP1 can promote mammary epithelial cell proliferation by enhancing hedgehog and NF-κB signaling to promote macrophage inflammatory responses [16]. Besides, AEBP1 is essential in modulating energy homeostasis by negatively regulating adipose tissue phosphatase and tensin homolog (PTEN) levels [17]. Aside from its role in normal physiological processes, AEBP1 plays a number of regulatory roles in physiology, particularly in cancer. For instance, AEBP1 promotes epithelial-mesenchymal transition (EMT) by regulating its downstream target gene, BMP4, which promotes papillary thyroid cancer development [18]. Furthermore, AEBP1 activation of PI3K/AKT signaling enhances the proliferation and spheroid production of glioblastoma (GSC) [19]. The carboxypeptidase CPZ (ID: 8532) belongs to the N/E subfamily and comprises a cysteine-rich domain (CRD) that removes basic amino acids from proteins’ carboxyl terminus [20]. According to reports, CPZ shares homology with Wnt receptors, suggesting it may regulate Wnt signaling [21]. Even though AEBP1 and CPZ have been studied in some pathophysiological processes, the manner in which they interact with the Wnt signaling pathway in GC is still unknown.

Due to limitations in assessing gastric cancer prognosis and Wnt signaling’s importance in its development and progression, our study aims to identify Wnt signaling-related prognostic signature genes for gastric cancer patients. After identifying these prognostic genes, including CPZ, CTHRC1, DKK1, EGF, and GPC3, we will explore their specific impact on gastric cancer progression, as well as the molecular mechanisms underlying it, particularly the CPZ. In this research, we aim to detect the underlying mechanisms of AEBP1 and CPZ in GC, and we conduct several functional experiments to verify the key roles of the transcription factor AEBP1 of CPZ in GC development. Understanding how these genes drive GC progression and metastasis is crucial as it may provide new strategies or targets for treatment.

## Materials and Methods

### Cell culture and transfection

At the Shanghai Institute of Biochemistry and Cell Biology, we obtained the immortalized gastric epithelium cell line GES-1 as well as the human GC cell lines AGS, HGC27, MGC-803, MKN28, and MKN45. Short tandem repeat fingerprinting was used to authenticate all cells for mycoplasma contamination. These cells were maintained in RPMI-1640 medium (SH30027.FS, HyClone, South Logan, UT, USA) enriched with 10% fetal bovine serum (FBS) (10099-141, Gibco, Grand Island, NY, USA) and 1% penicillin-streptomycin (No. P1400, Solarbio, Beijing, China) under conditions of 37°C and 5% CO_2_. Quercetin (849061-97-8, Sigma-Aldrich, St. Louis, MO, USA), a specific Wnt signaling pathway inhibitor, was administered at a concentration of 15 μM for 12 h. GenePharma (Shanghai, China) synthesized short-hairpin RNA plasmids (GV493) that target AEBP1 (shAEBP1-1, shAEBP1-2, and shAEBP1-3), as well as a non-targeting sequence (shScramble). The sequences were listed in Table S1. Lipofectamine 3000 reagent (L3000150, Life Technologies Corporation, Gaithersburg, MD, USA) was used to conduct transfection as described in the manufacturer’s protocols. Briefly, 4 × 10^5^ cells per well were added into six-well plate and when cells were grown to 70% to 90% density, 2.5 μg RNA and 5 μL Lipofectamine 3000 reagent were mixed at room temperature and added into each well after 20 min. A packaging plasmid mix and the GV493 vector were transfected into HEK293T cells to generate lentiviral vectors. The pTSB-CMV-AEBP1 vector and pTSB-CMV-FLAG-AEBP1 vector were purchased from Sangon Biotech (Shanghai, China). The AEBP1 cDNAs were PCR-amplified from HGC27 cells and subsequently subcloned into the vectors. Here are the primer sequences for AEBP1: forward AGACCACGCCATCTTCCG, reverse CCTTGTTGTTCTCCCACTCG. Table S2 lists the other primer sequences used for qRT-PCR in our research ().

### GC patients and tissue collection

GC and adjacent normal tissue (surgical margin ≥5 cm) samples were collected from 10 patients diagnosed with GC between January 2021 and December 2022 at the First Affiliated Hospital of Anhui Medical University, Hefei, China. Patients with gastric cancer who were included in this study had to be diagnosed with gastric cancer by pathological examination and had not previously received surgery, chemotherapy, or radiotherapy related to the treatment of gastric cancer. Regulatory approval was obtained from the ethics committee of the First Affiliated Hospital of Anhui Medical University with the number KY2023014. The Declaration of Helsinki was followed when collecting all samples.

### qPCR and western-blotting

An extraction of total RNA from gastric cancer tissues was carried out using Trizol reagent (#15596018, Life Technologies, Gaithersburg, MD, USA). We synthesized cDNA using the cDNA Synthesis SuperMix kit (#6215B, TaKaRa, Tokyo, Japan) and determined the relative levels of RNA expression using the SYBR-Green Real-Time Master Mix (#QPK-201, Toyobo, Osaka, Japan) and Lightcycler 96 real-time PCR system (Roche, Basel, Switzerland). Data normalization was done against β-actin.

A radioimmunoprecipitation assay (RIPA) lysis buffer (#P0013E, Beyotime Biotechnology, Shanghai, China) mixed with protease inhibitors (#45-4693132001, Roche, Basel, Switzerland) was used to extract the total protein. Protein quantification was done using the BCA™ Protein Assay Kit (23227, Pierce, Rockford, IL, USA). The Bio-Rad Bis-Tris Gel system was employed for western-blotting. Briefly, the proteins were separated by sodium dodecyl sulfate polyacrylamide gel electrophoresis (SDS-PAGE) and then transferred onto PVDF membranes (#FFP39, Beyotime Biotechnology, China) activated by methanol. The membranes were subsequently blocked with blocking buffer (#P0023B, Beyotime Biotechnology, China) at room temperature for 1 h. After incubated with the primary antibodies at 4°C overnight, the membranes were then washed and incubated with horseradish peroxidase-labeled secondary antibodies for 1 h at room temperature. For visualization, the Bio-Rad ChemiDoc^TM^ XRS system was used, and band intensities were quantified using Image Lab^TM^ Software (version 6.1, Bio-Rad, Hercules, CA, USA). We used the following antibodies: anti-CPZ (#PA5-103708, Thermo Fisher Scientific, Waltham, MA, USA, 1:2000), anti-AEBP1 (#sc-271374, Santa Cruz Biotechnology, Santa Cruz, CA, USA, 1:500), anti-TCF3 (#sc-166411, Santa Cruz Biotechnology, USA, 1:500), anti-β-catenin (#84441, Cell Signaling Technology, Danvers, MA, USA, 1:1000), anti-pGSK3β (#5558, CST, USA, 1:1000), anti-GSK3β (#12456, CST, USA, 1:1000), anti-Vimentin (#10366-1-AP, Proteintech, Wuhan, China, 1:5000), anti-TIMP2 (#sc-21735, Santa Cruz Biotechnology, USA, 1:500), anti-COL8A1 (#ab236653, Abcam, Cambridge, MA, USA, 1:5000), anti-Flag (#66008-4-Ig, Proteintech, China, 1:20000), anti-H3 (#68345-1-Ig, Proteintech, Wuhan, China, 1:10000), anti-β-actin (#66009-1-Ig, Proteintech, Wuhan, China, 1:30000), and anti-GAPDH (#60004-1-Ig, Proteintech, Wuhan, China, 1:200000).

### Co-immunoprecipitation assay

Cells were subjected to incubation with immunoprecipitation lysis buffer (#87787, Thermo Fisher Scientific, USA) on ice for a duration of 30 min. The lysates from these cells, containing a total protein amount of 1 mg, were then incubated with primary antibodies (2 µg) for TCF3, AEBP1, and FLAG overnight at 4°C. The cell lysates were then incubated at 4°C for six hours with protein A/G beads (#sc-2003, Santa Cruz, CA, USA). The normal mouse IgG (#sc-2025, Santa Cruz Biotechnology, USA) was utilized as a negative control. Post the bead washing step, the protein samples were subjected to analysis either through mass spectrometry or via western-blot analysis.

### Immunohistochemistry

The GC and adjacent normal tissues were subjected to immunohistochemical analysis. Tissues were paraffin-embedded and sectioned at 4 μm. At 4°C overnight, sections were stained with anti-AEBP1 (#sc-271374, Santa Cruz Biotechnology, USA, 1:400). Then the sections were stained in streptavidin-biotin-peroxidase complex with diaminobenzidine (S911, Thermo Fisher Scientific, USA) at room temperature for 20 min. Following three 1 × PBS washes, DAB solution (G1212-200T, Thermo Fisher Scientific, USA) was introduced and left to react in the dark at ambient temperature for half an hour. After washing twice with PBS, the color development was terminated and observed sections under a microscope (Olympus, IX73P2F, Tokyo, Japan).

### Chromatin immunoprecipitation (ChIP) and luciferase reporter assay

An Enzymatic Chromatin IP Kit (#9005, Cell Signaling Technology, Danvers, MA, USA) was used to perform the ChIP assay for AEBP1. To obtain DNA fragments, we sonicated HGC27 cells lysates for 20 s with 30-s intervals and repeated 15 times using a sonicator (120 W, 20 kHz) (#53052, Active Motif, USA) with its 25% power, and then incubated them with micrococcal nuclease. Antibodies against AEBP1 (#sc-271374, Santa Cruz Biotechnology, USA) and normal mouse IgG (#sc-2025, Santa Cruz Biotechnology, USA) were used for immunoprecipitation. Specific primers were used for semi-quantitative PCR analysis of the ChIP products (Table S3) followed by gel electrophoresis. Photographs were taken with a digital imaging system (Tanon, Shanghai, China).

We used the dual luciferase reporter assay system (#E1910, Promega, Madison, WI, USA) recommended by the manufacturer’s protocol to perform luciferase assays on 293T cells. Normalization was achieved through co-transfection with a Renilla plasmid. Site-directed deletion mutagenesis (mut-AEBP1 motif 1: ATTTC Deletion; mut-AEBP1: CAAAT Deletion) was constructed according to the instruction of Fast Mutagenesis Kit (#4992901, Tiangen Biotech, Beijing, China).

### Migration and invasion assays

The migration and invasion abilities of HGC27/MKN28 cells were assessed using 24-well Transwell chambers (3407, Corning Incorporated, Corning, NY, USA). Post-transfection (48 h), 5 × 10^4^ cells were inoculated in the upper chamber containing 200 μL complete medium, and in the lower chamber, 600 μL complete medium was inoculated. BD Matrigel^TM^ Matrix (#354234, BD Biosciences, San Jose, CA, USA) was coated onto the upper chamber for invasion assays. After incubation for 48 h, the chambers were collected and washed with 1 × PBS, then wiped with a cotton swab to remove any remaining cells. Then the cells were fixed with methanol for 30 min (#200-659-6, Sigma-Aldrich, St. Louis, MO, USA), and then stained with 0.1% crystal violet for 30 min (# 548-62-9, Merck, Germany). We then washed the chambers three times with 1 × PBS and counted them under a microscope (Olympus, IX73P2F, Japan).

### Bioinformatics analysis

Co-expression analysis based on Pearson correlation was performed on gene expression data. The R package ggplot2 (version 3.3.6) was used to plot Heatmaps, based on log_2_(TPM + 1) values, with rows normalized relative to their mean values. For gene sets that were co-expressed or enriched in the same functional annotations, transcription factor predictions were made through the ChEA3 platform (https://maayanlab.cloud/chea3/, accessed on 12 March 2022) [22].

TCGA-STAD RNA-seq data analysis was performed via the GEPIA2 platform (http://gepia2.cancer-pku.cn, accessed on 12 March 2022) [23]. Single-cell tSNE distribution plots were computed on single-cell RNA-seq data GSE150290 (https://www.ncbi.nlm.nih.gov/geo/query/acc.cgi?acc=GSE150290, accessed on 12 September 2022) through the R package Seurat workflow (version 3.1.1) [24]. Using the Human Protein Atlas (HPA) website (https://www.proteinatlas.org, accessed on 13 April 2022), we obtained images of GC tissues and normal gastric tissues stained with the same antibody and bar graphs showing gene expression levels in normal gastric tissues [25]. The R package DESeq2 (version 1.44.0) was used to identify differentially expressed genes between Normal and Tumor groups in TCGA-STAD RNA-seq data, [26] followed by Benjamini and Hochberg correction for multiple comparisons.

For univariate analysis of disease prognosis, the Cox proportional hazards regression model was used. The least absolute shrinkage and selection operator (LASSO) regression algorithm, implemented using the R package glmnet (version 3.6), was used to identify prognostic risk signature genes [27].

The R package clusterProfiler (version 3.2.11) was used to conduct enrichment analyses of the Kyoto Encyclopedia of Genes and Genomes (KEGG) and the Gene Ontology (GO) [28]. Gene set enrichment analysis (GSEA) was performed using the MSigDB dataset c2.cp.all.v2022.1.Hs.symbols.gmt [All Canonical Pathways] [29] and the R package clusterProfiler (version 3.2.11).

Potential AEBP1 interacted proteins were searched in two protein interaction databases: Biological General Repository for Interaction Datasets (BioGRID, https://thebiogrid.org/, accessed on 15 April 2022) [30] and Search Tool for Recurring Instances of Neighbouring Genes (STRING, https://cn.string-db.org/, accessed on 20 April 2022) [31]. With a medium confidence score of 0.400, a Protein-protein interaction (PPI) network centered on AEBP1 was generated using the STRING website platform.

Utilizing the HDock server (http://hdock.phys.hust.edu.cn/, accessed on 22 April 2022), molecular docking simulations were conducted to see how AEBP1 and TCF3 interact directly [32]. HDock employs a hybrid algorithm for molecular docking and molecular structure refinement, and the docking states of the two protein structures were visualized using Pymol2 software (https://pymol.org/, accessed on 12 November 2024, Schrodinger, Manhattan, NY, USA).

### Intraperitoneal metastasis assays

All the animal studies we performed have obtained approval from the Institutional Animal Care and Use Committee of Anhui Medical University (LLSC20232267), and followed the ARRIVE guidelines. The female BALB/c-Nude mice (1 week) were purchased from GemPharmatech (D000521, Nanjing, China) and housed in specifically pathogen-free (SPF) conditions with 12/12 h of light and dark cycles at 25°C. 1 mL 1 × 10^6^ of HGC27 cells post-treatment were intraperitoneally injected into female BALB/C nude mice (6 weeks, 18–22 g). After 4 weeks, euthanasia was induced using 3%–5% concentrations of isoflurane, followed by cervical dislocation of the mice, and the intraperitoneal tumor nodules were counted.

### Statistical analysis

GraphPad 8.0 software (San Diego, USA) was used to analyze the data and present it as mean + standard deviation (SD). Statistical significance was determined using the student’s *t*-test and one-way ANOVA with Bonferroni correction. The *p*-value for statistical significance was less than 0.05.

## Results

### Identification of gastric cancer prognostic signature genes

**Figure 1:**
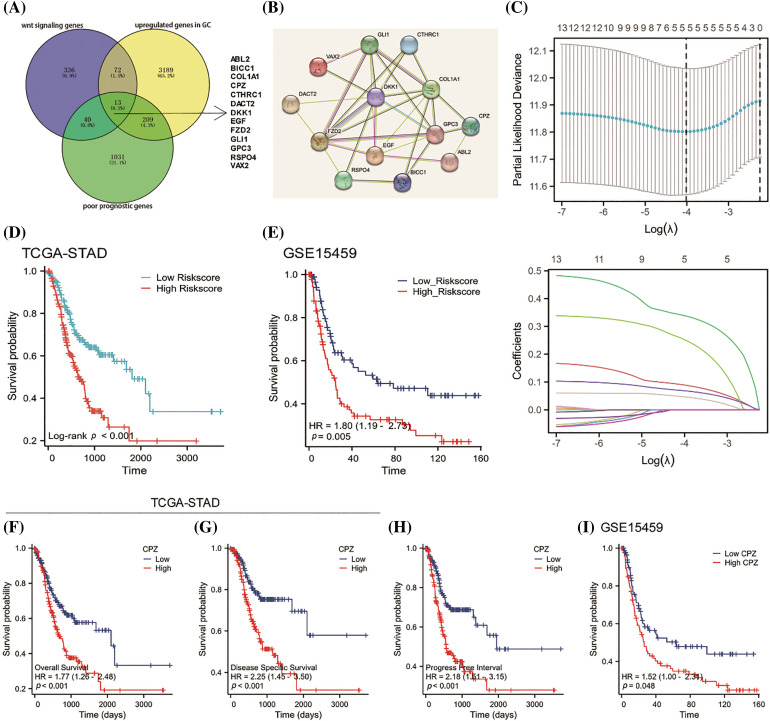
Identification of prognostic gene signature and survival analysis. (A) A high expression of Wnt signaling pathway-related genes was found in GC tissues and was associated with a poor prognosis. (B) The LASSO regression analysis identifies prognostic signature genes associated with the Wnt signaling pathway in GC patients. (C) Identification of prognostic signature genes in GC patients. (D) A risk score based on signature genes predicts poor prognosis of GC patients in the TCGA-STAD database. (E) A risk score based on signature genes predicts poor prognosis of GC patients in the GSE15459 database. (F–H) CPZ expression predicted the overall survival (F), disease-free survival (G), and progression-free interval (H) of GC patients in the TCGA-STAD database. (I) CPZ expression predicted the overall survival of GC patients in the GSE15459 dataset.

Given the intimate link between the Wnt signaling pathway and gastric cancer progression, we attempted to enhance the prognostic prediction of GC by conducting an analysis of prognostic signature genes and further investigating the mechanisms related to these genes. The analysis of TCGA-STAD RNA-seq data identified 13 Wnt signaling pathway-related genes that are both overexpressed in gastric cancer tissues (Log_2_Fold Change (FC) > 0.585, adjust. *p* < 0.05) and associated with poor prognosis (hazard ratio (HR) > 1 and *p* < 0.05 based on Cox regression model) (Fig. 1A). These gene-encoded proteins form an interactive regulatory network (Fig. 1B). Using LASSO regression analysis (Fig. 1C), we examined five signature genes, whose risk scores are capable to predicting the prognosis of GC accurately: Risk Score = 0.3392 × CPZ + 0.0894 × CTHRC1 + 0.0468 × DKK1 + 0.2534 × EGF + 0.0687 × GPC3 (Fig. 1D). This method helps in identifying the most relevant genes that contribute to the model by imposing a penalty on the size of the coefficients. The coefficients in the risk score formula reflect the strength and nature of the relation of each gene with GC prognosis. GEO data GSE15459 from the GEO database was used to validate the finding from TCGA-STAD (https://www.ncbi.nlm.nih.gov/geo/query/acc.cgi?acc=GSE15459, accessed on 12 February 2023) (Fig. 1E).

Among these five signature genes, CPZ has been less studied in gastric cancer, prompting us to analyze its prognostic impact. Patients with GC who expressed high levels of CPZ had a poor prognosis, including overall survival (Fig. 1F), disease-specific survival (Fig. 1G), progress-free survival (Fig. 1H) in TCGA-STAD, and overall survival in GSE15459 (Fig. 1I).

### CPZ expression and co-expression network in gastric cancer tissues

**Figure 2:**
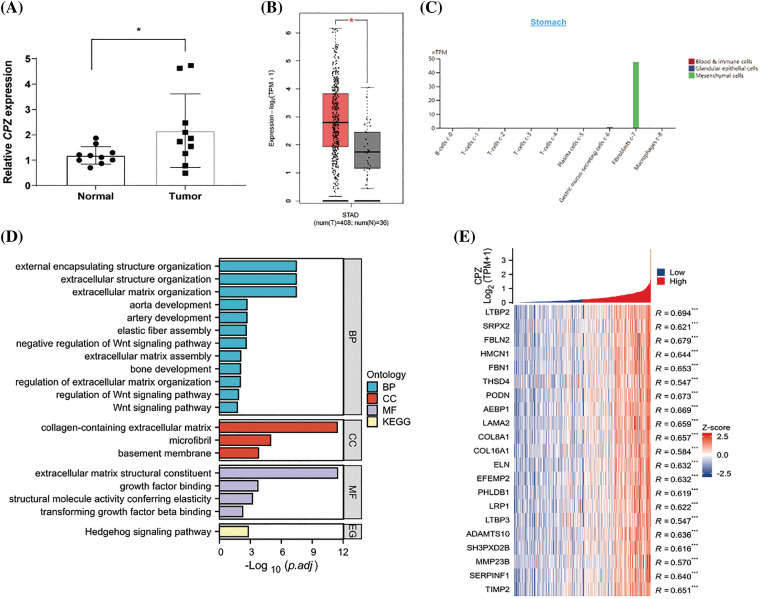

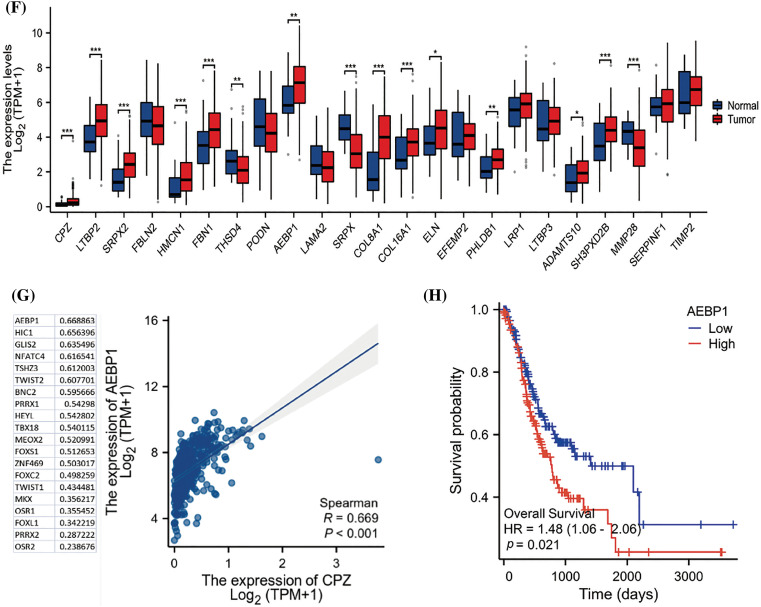
Expression of CPZ and its co-expressed genes in GC tissues. (A) In the clinical samples we collected, CPZ was more highly expressed in GC tissues than in paracancerous normal tissues. (B) There was a greater expression of CPZ in GC tissues compared to normal tissues in the TCGA-STAD dataset (GEPIA2 platform). (C) CPZ was specifically expressed in fibroblasts of normal gastric tissue. (D) Enriched functions in GO/KEGG by CPZ co-expressed genes. (E) Histogram showing expression levels of CPZ co-expressed genes. (F) Correlation between AEBP1 and CPZ expression. (G) Expression levels of ChEA3-predicted transcription factors in GC tissues and normal tissues (TCGA-STAD). (H) AEBP1 expression predicted the prognosis of GC patients. *p < 0.05; **p < 0.01; ***p < 0.001.

Given CPZ’s significant prognostic impact, we confirmed its overexpression in gastric cancer tissues using qPCR on our clinical patient samples (Fig. 2A,B). According to TCGA-STAD data, CPZ mRNA levels are higher in tissue from GC compared to normal tissue (Fig. 2B).

As reported, CPZ is a carboxypeptidase that is secreted and acts extracellularly (the optimum active pH is neutral) [20]. In addition to tumor cells, gastric cancer tissue also contains a variety of mesenchymal cells like fibroblasts, endothelial cells, and immune cells such as T and B cells. To clarify the cell specificity of CPZ, we searched the HPA database (https://www.proteinatlas.org, accessed on 11 May 2022). According to our findings, CPZ is mostly expressed in fibroblasts in normal gastric epithelium (Fig. 2C). Single-cell RNA sequencing data GSE150290 showed the gene SERPINF1 highly correlated with CPZ expression (R = 0.640, *p* < 0.001) was mainly expressed in fibroblasts in normal gastric tissue (Fig. S1A). In GC tissue, in addition to the expression of fibroblasts, SERPINF1 was also expressed in tumor cells, suggesting a similar expression pattern of CPZ (Fig. S1B).

To understand the function of CPZ, we further performed functional enrichment analysis of CPZ co-expressed genes. Co-expression analysis showed that the top 100 genes significantly positively correlated with CPZ expression were mainly enriched in extracellular matrix-related annotations, among which Biological Process (BP), Cellular Component (CC), and Molecular Function (MF) annotations that ranked first each were: “extracellular matrix structural constituent”, “extracellular matrix organization”, and “collagen-containing extracellular matrix”, separately (Fig. 2D). Most of the genes enriched in these annotations were strongly positively correlated with CPZ expression (R > 0.6, *p* < 0.001) (Fig. 2E). It is worth noting that in TCGA-STAD data, the expression levels of these co-expressed genes were higher than CPZ (Fig. 2F), suggesting that CPZ may be just a representative signature gene rather than a major gene in extracellular matrix regulation. The explicit data of correlation coefficients and *p*-values is listed in (Tables S4–5).

To identify the key transcription factor of these extracellular matrix regulation-related genes, we performed ChEA3 transcription factor prediction analysis on the CPZ co-expressed genes enriched in these extracellular matrix annotations and identified the top three transcriptional regulators PRRX1, AEBP1, and OSR1, of which AEBP1 and OSR1 potentially regulate the transcription of CPZ. Then, we performed ChEA3 transcription factor prediction analysis on the top 100 genes positively correlated with CPZ expression and found the top 3 transcription factors PRRX, PRRX2, and AEBP1. Both PRRX2 and AEBP1 potentially regulate the transcription of CPZ. It is worth noting that AEBP1 has the strongest correlation with CPZ expression among all the predicted transcription factors (R = 0.669, *p* < 0.001) (Fig. 2G). Compared with other predicted transcription factors, the expression level of AEBP1 was also the highest (Fig. S1C). Therefore, we speculate that AEBP1, as a transcription factor, is an initiating factor of the CPZ co-expression network and the expression of extracellular matrix-regulating genes in GC tissues.

Further, survival analysis and immune infiltration analysis corroborated the importance of AEBP1. Survival analysis also showed that AEBP1 expression was significantly associated with poor prognosis in GC patients (Fig. 2H).

Because AEBP1 is likely to be the upstream initiating factor of the CPZ co-expression network, we will further focus on the function of AEBP1.

### Expression of AEBP1 in gastric cancer tissues and cell lines

**Figure 3:**
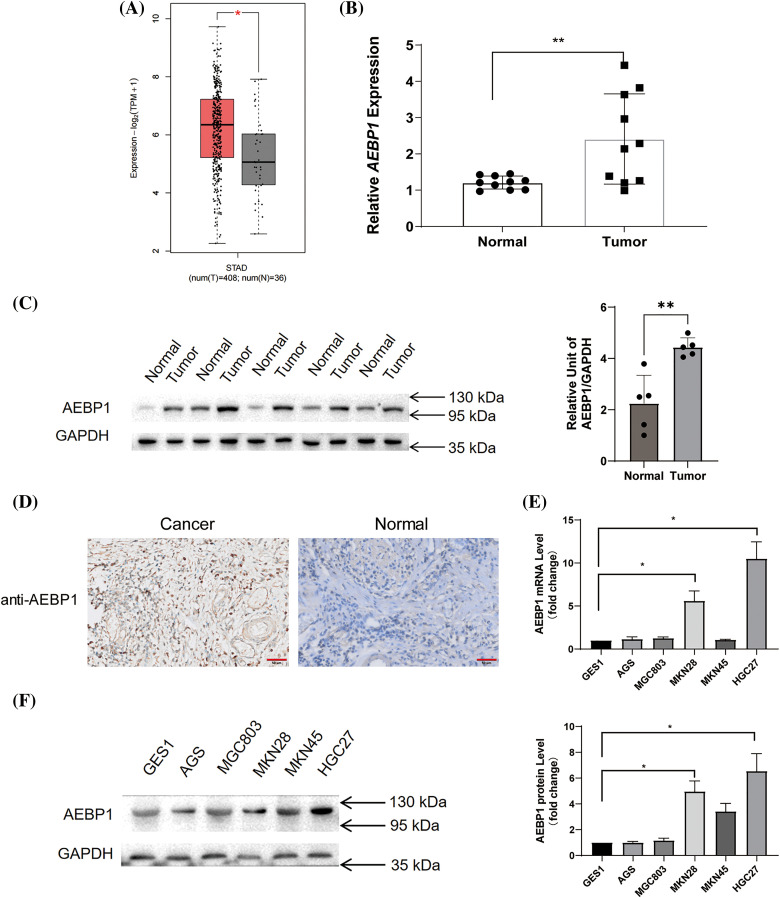
Expression of AEBP1 in GC tissues and cells. (A) In the TCGA-STAD dataset (GEPIA2 platform), AEBP1 was highly expressed in GC tissues compared with normal tissues. (B) The expression of AEBP1 in GC tissues was higher than in adjacent normal tissues in the clinical samples we collected. (C) In the clinical samples we collected, AEBP1 had higher protein levels in GC tissues than in paracancerous normal tissues. (D) According to clinical samples, AEBP1 is highly expressed in GC tissues but low in adjacent normal tissues. (E and F) Expression of AEBP1 in different GC cell lines (E: mRNA level, F: protein level). *p < 0.05; **p < 0.01.

Using the GEPIA2 platform, we analyzed TCGA-STAD data for expression of AEBP1, which showed higher expression in tumor samples than in normal samples (Fig. 3A). We then detected the AEBP1 mRNA and protein levels with clinical GC and paracancerous tissue samples collected by ourselves and verified its high expression in gastric cancer tissues (Fig. 3B,C). Next, we analyzed the expression of AEBP1-correlated gene BGN (R = 0.901, *p* < 0.001, Fig. S2A) in single-cell RNA-seq data, and found that BGN was expressed in fibroblasts of normal tissues, and when cancer occurred, it was mainly expressed in tumor cells (Fig. S2B). HPA database single-cell data showed that in normal gastric tissue, AEBP1 was mainly expressed in fibroblasts (Fig. S2C).

Immunohistochemistry of the HPA database (Fig. S2D) and our own tissue samples (Fig. 3D) showed the same results, that is, in normal tissues, AEBP1 was less expressed, while when cancer occurred, AEBP1 was highly expressed in GC tissues.

Different GC cell lines were analyzed to determine the expression levels of AEBP1. As we found, AEBP1 was highly expressed in GC cell lines HGC27 and MKN28, but not in gastric mucosal epithelial cells GES-1 or GC cells MKN45 and AGS. (Fig. 3E,F).

### AEBP1 affects the expression of a series of extracellular matrix-related genes and binds to the promoter region of CPZ to promote its transcription

**Figure 4:**
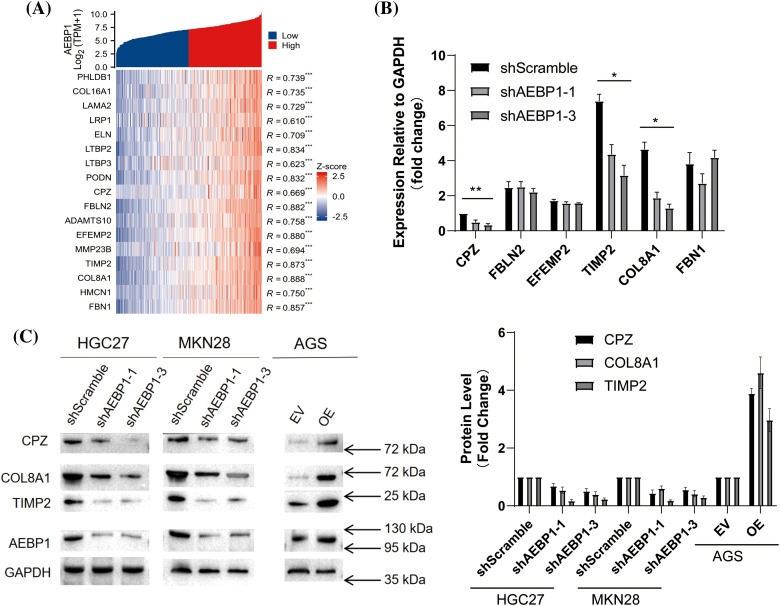
The influence of AEBP1 on the expression of its correlated genes. (A) Heat map of AEBP1 gene co-expression. (B and C) Changes of AEBP1-correlated genes after the knockdown or overexpression of AEBP1 (B: mRNA level, C: protein level). *p < 0.05; **p < 0.01; ***p < 0.001.

To further explore the effects of AEBP1 on gastric cancer cells, we established cell lines with stable knockdown of AEBP1 by shRNA or with the overexpression of AEBP1 (Fig. S3A–F). On this basis, we analyzed the expression of AEBP1-positively correlated genes (R > 0.85, *p* < 0.001) (Fig. 4A) and then detected the mRNA levels of AEBP1-correlated genes (Fig. 4B) showing the significant decrease of CPZ, COL8A1 and TIMP2 after knocking down AEBP1. Furthermore, our analysis of their protein levels (Fig. 4C) confirmed the changes in their mRNA levels (Fig. 4B).

**Figure 5:**
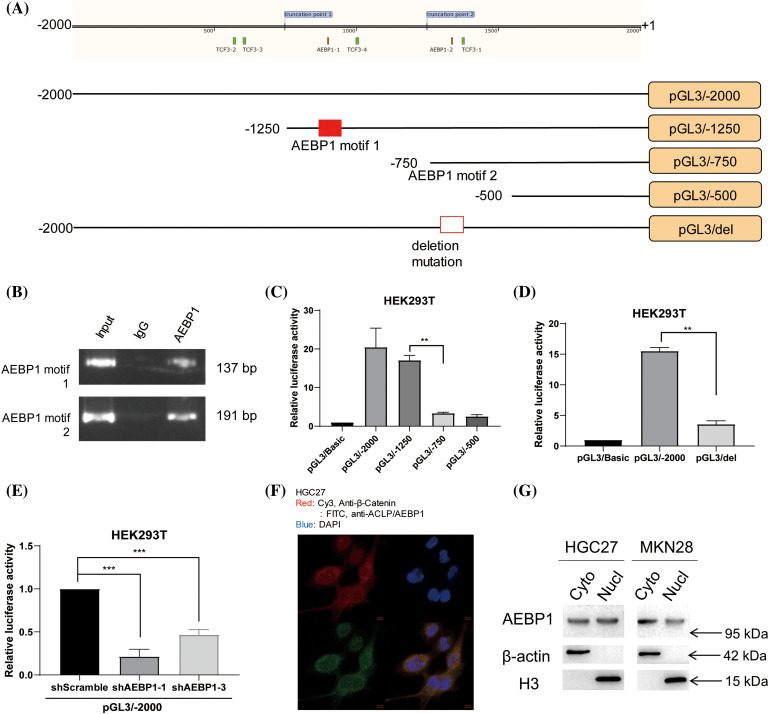
AEBP1 upregulates CPZ transcription in the nucleus by interacting with its promoter. (A) Schematic diagram of the CPZ promoter sequence and its truncated forms. (B) ChIP experiments show the binding of AEBP1 to the motif regions in the CPZ promoter. (C) The −1250 to −750 region of the promoter is a key binding region for AEBP1 to up-regulate gene expression. (D) The motif 1 bound by AEBP1 is a key region for the upregulation of gene expression. (E) The luciferase reporter was downregulated significantly by AEBP1 knockdown. (F) Immunofluorescence shows the cellular distribution of AEBP1 (red). (G) Western blot showing the nucleocytoplasmic distribution of AEBP1 after nucleocytoplasmic separation. *p < 0.05; **p < 0.01; ***p < 0.001.

It has been reported in the literature that AEBP1 binds to the motif GAAAT [33]. In order to clarify the role of AEBP1 in CPZ transcription regulation, we downloaded the 2000 bp promoter region upstream of the CPZ transcription start site and identified the AEBP1 motifs in the promoter (AEBP1 motif 1: ATTTC from −1098~−1102, AEBP1 motif 2: GAAAT from −662~−666) (Fig. 5A). We first verified the binding of AEBP1 on its corresponding motif regions by ChIP experiments (Fig. 5B). Then, by constructing and transfecting pGL3 plasmids containing different truncations of the promoter region (Fig. 5A), we identified the key region upstream of the transcription start site: the −750 to −500 region (Fig. 5C). Since this region contains an AEBP1-binding motif sequence, we further deleted and mutated the AEBP1-binding sequence (Fig. 5A), and found that the promoter activity was significantly reduced in the deletion mutation sequence pGL3/del, clarifying the necessity of AEBP1 binding in this region for transcription (Fig. 5D). To further confirm the effect of AEBP1 in the regulation of CPZ transcription, we knocked down AEBP1 in H293T cells with pGL3/-2000 Luciferase Reporter vector and found the downregulated luciferase activity (Fig. 5E). The above results indicated that AEBP1 is an important transcription factor for up-regulating CPZ.

As descripted in previous studies, AEBP1 can affect the inflammatory phenotype of macrophages and the epithelial-mesenchymal transition (EMT) phenotype of GC cells by promoting NF-κB or MAPK signaling pathways in the cytoplasm [13,14,34]. To clarify the spatial localization of AEBP1 in gastric cancer cells, by immunofluorescence experiment (Fig. 5F) and western-blot after nuclear and cytoplasmic separation (Fig. 5G), we found that in addition to the existence of AEBP1 in the cytoplasm, part of AEBP1 was localized in the nucleus. Therefore, it is possible for AEBP1 to directly affect the transcription of genes in the nucleus in addition to affecting signaling pathways in the cytoplasm.

### AEBP1 regulates gene transcription by interacting with TCF3

**Figure 6:**
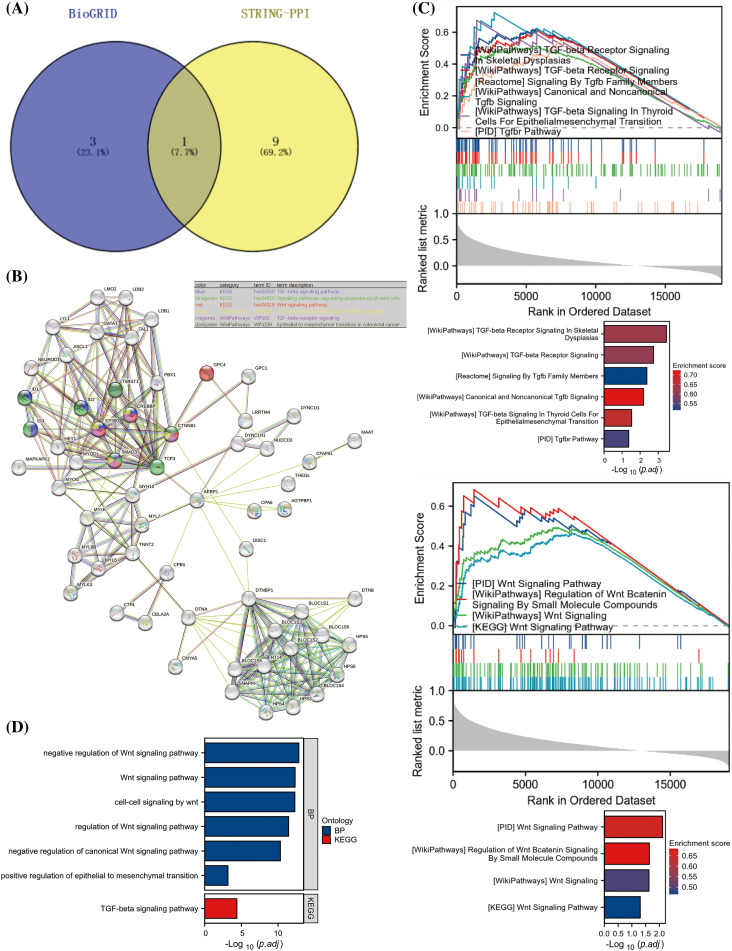
Prediction of AEBP1-interacting proteins and AEBP1-affected signaling pathways. (A) Intersection of AEBP1 interacting proteins in STRING-PPI and BioGRID databases. (B) AEBP1 STRING-PPI network and the Wnt signaling enriched proteins. (C) GSEA enrichment analysis according to AEBP1 correlation of genes. (D) The Wnt signaling level in GC cells was detected after AEBP1 was knocked down or overexpressed.

In order to understand how AEBP1 works in gene transcription regulation, we took the intersection of BioGRID and STRING databases and found that TCF3 and AEBP1 interact with high confidence. Since TCF3 is a transcription factor, we predicted that TCF3 interacted with AEBP1 in the nucleus, and they worked together to regulate downstream gene transcription (Fig. 6A). Additionally, we analyzed the STRING-PPI network of AEBP1 in terms of functional enrichment, and found that AEBP1 networks were enriched in TGF-βand Wnt signaling. Through its interaction with TCF3, AEBP1 is related to proteins enriched in TGF-/Wnt signaling pathways (Fig. 6B). We found that these enriched proteins, including EP300, CREBBP (CREB binding protein), SMAD3 (SMAD family member 3), and CTNNB1 (catenin beta 1), were all proteins related to gene transcription: EP300 and CREBBP were acetyltransferases that promote the transcriptional activity of transcription factors (including TCF3); SMAD3 was a key transcription factor downstream of the TGF-β signaling pathway; TCF3 and CTNNB1 (encoding β-catenin) were key transcription factors downstream of the Wnt signaling. In this regard, they may cooperate with AEBP1 to regulate transcription.

To detect the AEBP1-related cell signaling pathways, we sorted the genes in the TCGA-STAD database according to their expression correlation with AEBP1 and then performed the GSEA enrichment analysis related to the signaling pathway. The result showed that both TGF-β and Wnt signaling pathway was significantly enriched in the AEBP1-positively correlated gene region, suggesting that AEBP1 was likely to enhance the transcription of TGF-β and Wnt-signaling related genes (Fig. 6C). Furthermore, the top 100 genes positively correlated with AEBP1 were analyzed for GO/KEGG enrichment, and found that the positively correlated genes were also significantly enriched in the TGF-β and Wnt signaling, with Wnt signaling pathway enrichment being the highest (Fig. 6D).

**Figure 7:**
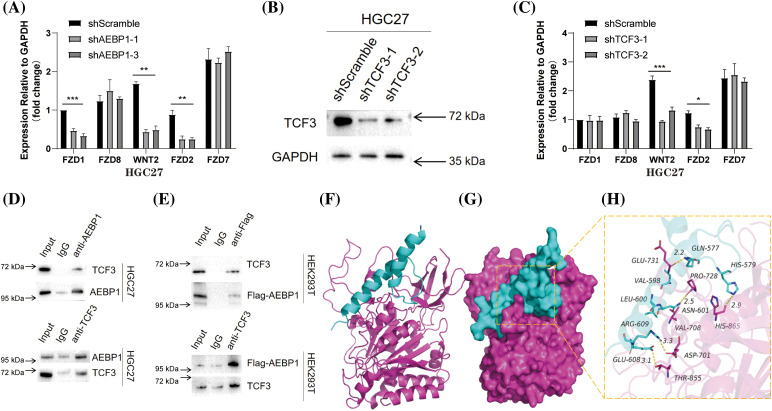
Regulation of genes related to Wnt signaling pathway by AEBP1 and TCF3 in HCG27 cells stimulated with Wnt activator CHIR99021. (A) qPCR analysis of gene transcription after AEBP1 knockdown. (B) Verification of TCF3 knockdown in HGC27. (C) qPCR analysis of gene transcription after TCF3 knockdown. (D) Co-IP showed that AEBP1 and TCF3 interacted in HGC27 cells stimulated with the Wnt activator CHIR99021. (E) Overexpression of Flag-AEBP1 in HEK293T can co-immunoprecipitate with TCF3. (F) The backbone of the protein was rendered in a tube and colored in cyan (TCF3) and red (AEBP1). (G) TCF3 and AEBP1 protein is rendered by the surface. (H) The detailed binding mode of TCF3 with AEBP1. The yellow dash represents the hydrogen bond. *p < 0.05; **p < 0.01; ***p < 0.001.

The intersection of core genes enriched in the Wnt signaling pathway was selected to verify whether AEBP1 is involved in transcription downstream of Wnt signaling. In the AEBP1 knockdown GC cell line HGC27, the Wnt/β-catenin signaling activator CHIR99021 (GSK-3β inhibitor, 3μM) was used to stimulate the cells, and then the transcription was detected by qPCR. It was found that knocking down AEBP1 significantly inhibited the transcription of some Wnt signaling (Frizzled Class Receptor 1 (FZD1), WNT2, FZD2) genes (Fig. 7A). Similarly, in TCF3 knockdown HGC27 cells (Fig. 7B) treated with the Wnt activator CHIR99021, the transcription levels of Wnt signaling pathway genes WNT2 and FZD2 were significantly reduced (Fig. 7C), suggesting a synergistic effect between AEBP1 and TCF3.

Therefore, we detected the interaction between TCF3 and AEBP1 by co-IP in GC cell line HCG27 stimulated by Wnt activator CHIR99021, proving that they interacted after activation of the Wnt signaling (Fig. 7D). On the other hand, in exogenous experiments, we overexpressed Flag-tagged AEBP1 protein in HEK293T cells and found that it could also interact with TCF3 (Fig. 7E). Molecular docking simulation further suggested the direct interaction between AEBP1 and TCF3 (Fig. 7F–H): the binding score of TCF3 and AEBP1 protein was −239.32 kcal/mol, which was relatively stable; the binding sites of AEBP1 and TCF3 protein included PHE-97, ILE-121, ASN-205, PRO-191, ARG-183, GLU-131, and ASN-102 amino acid residues on TCF3, VAL-331, ALA-347, ASP-346, ARG-381, ASP-373, ARG-337, and GLN-556 amino acid residues on AEBP1. Several interactions can occur between TCF3 and AEBP1 residues, such as salt bridges (ARG-609: ASP-701), hydrogen bonds (GLN-577: GLU-731, HIS-579: HIS-865, ASN-601: PRO-728, GLU-608: THR-855), hydrophobic bonds (VAL-598: PRO-728, LEU-600: VAL-708) and other interactions, these interactions can effectively improve the stability of the TCF3 and AEBP1 protein complex.

### AEBP1 cooperates with the Wnt signaling to regulate the transcription of the VIM gene

**Figure 8:**
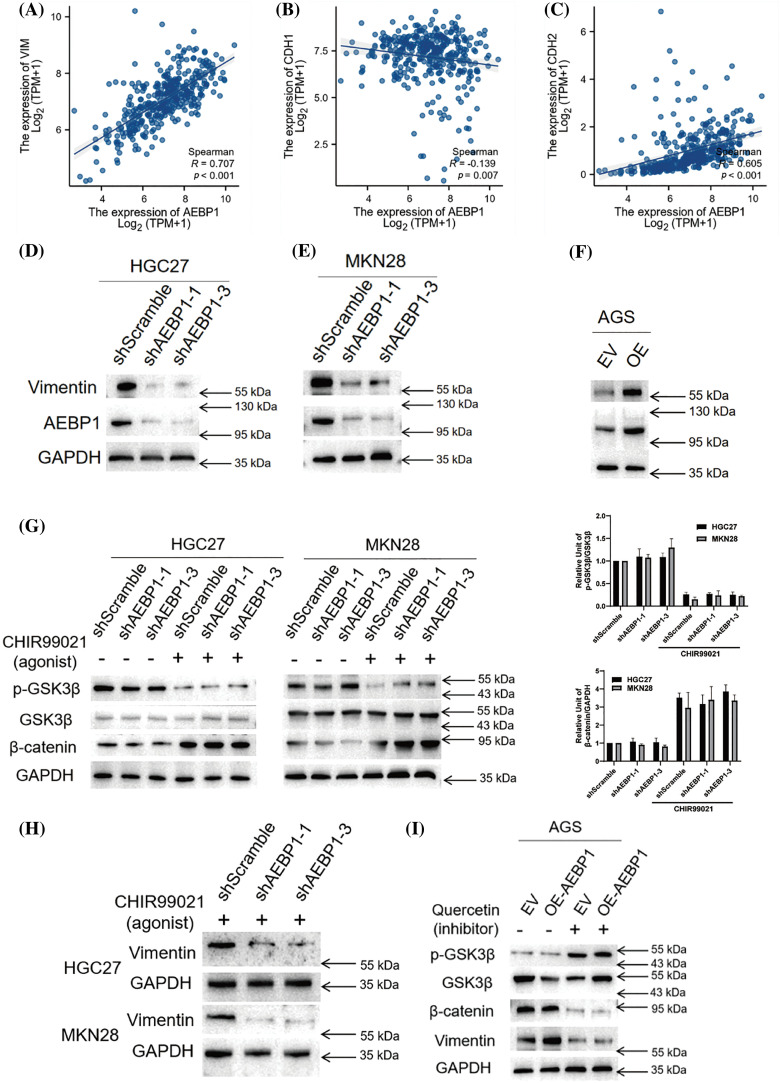
AEBP1 is responsible for regulating VIM gene expression. (A–C) Correlation of AEBP1 and EMT marker genes in TCGA-STAD database. (D–F) By knocking down or overexpressing AEBP1 in GC cell lines, VIM expression can be inhibited or increased. (G–H) Knockdown of AEBP1 does not affect the activation of Wnt signaling (G) but inhibit vimentin expression (H). (I) AEBP1 overexpression significantly promotes the expression of VIM only when the Wnt signaling pathway is not inhibited.

The Wnt signaling is recognized for enhancing the EMT characteristics of cancer cells, thereby facilitating their invasion and migration [35]. An analysis of TCGA-STAD data revealed a great correlation between AEBP1 expression and EMT markers (E-cadherin/CDH1, N-cadherin/CDH2, Vimentin/VIM), especially highly correlated with the expression of VIM (R = 0.707, *p* < 0.001, Fig. 8A–C), suggesting its direct transcriptional regulation on VIM.

First, we detected the changes in VIM expression level after AEBP1 knockdown in HGC27 and MKN28 cells, and found that VIM was significantly reduced (Fig. 8D,E). After overexpressing AEBP1 in AGS cells, VIM was significantly upregulated (Fig. 8F), indicating that AEBP1 is a key transcription factor for VIM expression.

For clarification of the cause of AEBP1 upregulation on VIM transcription in the nucleus *vs*. changes in Wnt signaling in the cytoplasm, after stable knockdown or overexpression of AEBP1, the Wnt signaling activation level in GC cells was detected. It was found that AEBP1 knockdown and overexpression did not significantly change the activation level of the Wnt signaling (Fig. 8G).

To explore the necessity and causation of AEBP1 in Wnt signaling pathway-upregulated VIM expression, we tested the effect of knocking down AEBP1 on Vimentin expression after activating the Wnt signaling pathway (Fig. 8H), and whether overexpression of AEBP1 can reverse the decline of Vimentin after inhibiting Wnt signaling pathway in HGC27 cells (Fig. 8I). Firstly, the result showed that the upregulation of VIM transcription by activated Wnt signaling pathway depended on the synergistic effect of AEBP1 (Fig. 8H), indicating the necessity of AEBP1. However, the overexpression of AEBP1 failed to reverse the decline of Vimentin after Wnt signaling pathway inhibitor Quercetin (40 μM) treatment (Fig. 8I), suggesting AEBP1 is an insufficient condition for VIM transcription. Therefore, the upregulation of VIM by AEBP1 requires the cooperative participation of β-catenin downstream of the Wnt signaling. However, further investigation is needed to determine whether AEBP1 or TCF3 are necessary conditions for VIM transcription after activation of the Wnt signaling by CHIR99021.

### Wnt pathway inhibitor quercetin synergizes with AEBP1 silencing to inhibit gastric cancer cell metastasis

Wnt signaling inhibitors have shown certain effects in the treatment of tumors, especially in the inhibition of metastasis of solid tumors [36]. Our above results suggest that since AEBP1 cooperates with key transcription factor TCF3 downstream of the Wnt signaling in GC, the knockdown of AEBP1 may enhance the inhibitory effect of Wnt pathway inhibitors on tumor metastasis.

**Figure 9:**
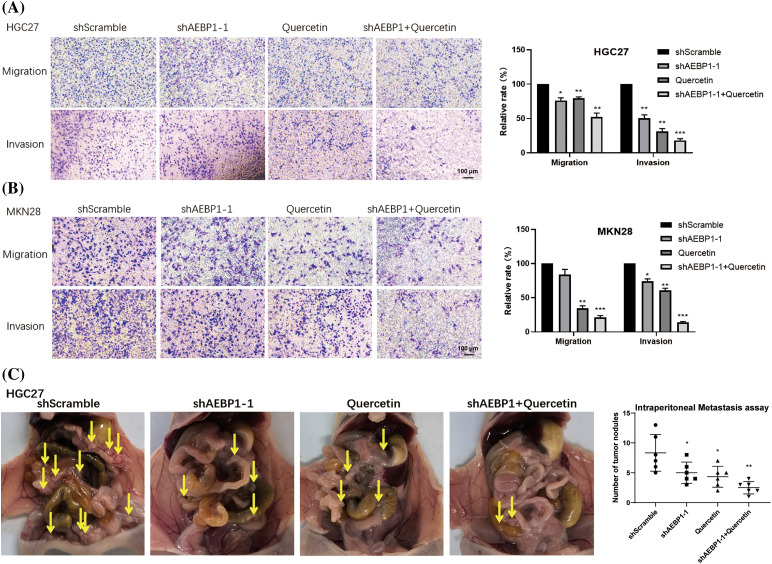
AEBP1 knockdown synergistically promoted the inhibitory effects of Wnt signaling pathway inhibitors on the migration and invasion of GC cell lines. (A) Synergistic inhibition of gastric cancer cell migration and invasion in HGC27. (B) Inhibition of migration and invasion of GC in MKN28 with synergistic effects. (C) AEBP1 shRNA and Wnt signaling pathway inhibitors synergistically inhibited the number of metastatic nodules (the yellow arrows) of HGC27 in the nude mouse peritoneal metastasis model. *p < 0.05; **p < 0.01.

Thus, we tested the effect of knocking down AEBP1 in combination with Quercetin, a Wnt pathway inhibitor, on gastric cancer cell invasion and migration. AEBP1 shRNA together with Quercetin were more effective at inhibiting migration and invasion of HGC27 and MKN28 cells (Fig. 9A,B).

*In vivo*, we utilized a mouse peritoneal metastasis model and found that the combination of AEBP1 stable knockdown and Wnt pathway inhibitor Quercetin had the most significant inhibitory effect on the peritoneal intestinal metastasis of gastric cancer cell HGC27 (Fig. 9C).

## Discussion

The Wnt/-catenin signaling is closely associated with the occurrence and metastasis of tumors, and their inhibitors are gradually being used in the treatment of GC [36]. The prognosis of gastric cancer patients is still unsatisfactory. In view of the relationship between the Wnt signaling pathway and gastric cancer, we identified the prognostic five genes signatures related to the Wnt pathway in this study, including CPZ, CTHRC1, DKK1, EGF, and GPC. The risk score based on their expression levels can well predict the prognosis of gastric cancer patients.

**Figure 10:**
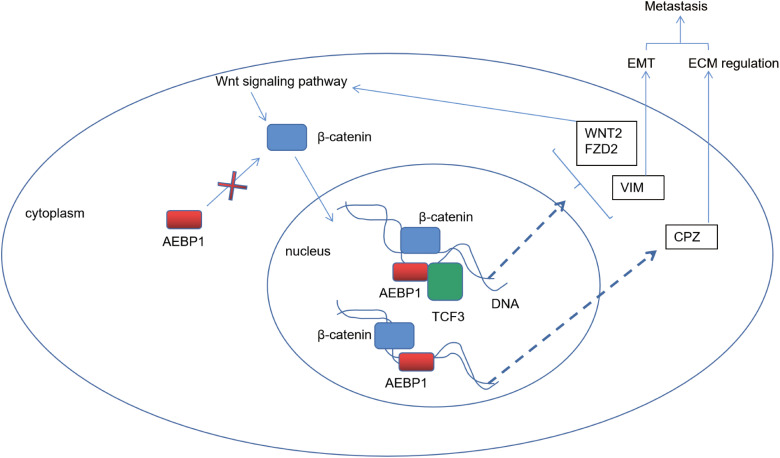
AEBP1 workflow diagram. The upstream CPZ (Carboxypeptidase Z) transcription factor AEBP1 (Adipocyte enhancer-binding protein 1) and downstream Wnt signaling pathway transcription factor TCF3 (Transcription factor 3) synergistically interact in the nucleus to enhance the expression of genes co-expressed with CPZ and those associated with the Wnt signaling pathway, including WNT2/FZD2 (Wnt family member 2/Frizzled class receptor 2) and VIM (Vimentin), an epithelial-mesenchymal transition marker. This process facilitates invasion, migration, and malignant metastasis of GC.

Among the five-gene signatures, CTHRC1, DKK1, EGF, and GPC3 have been studied in GC. CTHRC1, which is found in high levels in various human solid tumors including gastric cancer, has been shown to have a significant association with the infiltration of macrophages [37]. DKK1, a secretory antagonist that interacts with the Wnt coreceptor LRP5/6, thereby reducing cellular sensitivity to canonical Wnt ligands, has been identified as a factor that can enhance the recurrence of gastric cancer [38]. The existence of EGF in GC has been found to be linked with the extent of gastric wall invasion and lymph node metastasis [39]. As a result of binding to Wnt signaling proteins and growth factors, GPC3 plays an important role in Hepatocellular Carcinoma progression [40]. For gastric cancer, GPC3 can promote its metastasis [41]. However, the relationship between CPZ and GC has not been fully studied. We found that it represented a series of extracellular matrix-regulating genes, and was regulated by a key upstream transcription factor AEBP1. AEBP1 and TCF3 downstream of the Wnt/β-catenin signaling pathway interact and synergistically promote the expression of a series of genes involved in the regulation of extracellular matrix and epithelial-mesenchymal transition, including CPZ, to promote malignant metastasis of GC (Fig. 10).

In this study, we found that as a result of the interaction of AEBP1 and TCF3 in the nucleus, some genes downstream of the Wnt pathway were up-regulated synergistically. TCF3 (also known as E2A or ITF1) belongs to the TCF/LEF family and plays an important role in embryonic stem cells, immune cell development, and Wnt signaling as a transcription factor [42]. For example, TCF3 can interact with HLH (helix-loop-helix) domain-containing proteins E12 and E47 to regulate lymphocyte differentiation. As the Wnt signaling pathway is activated, the stability of β-catenin plays a key role. Then TCF3, as part of the TCF family, associates with β-catenin, which is stabilized in the cytoplasm during Wnt signaling activation and translocates to the nucleus. After β-catenin binds to TCF3, they jointly promote the expression level of Wnt-targeted genes [43,44]. If the Wnt signaling is inactivated, TCF3 may exist in an inhibitory form, repressing the expression of certain genes [43]. The experimental analyses showed that AEBP1, an upstream transcription factor of CPZ, and TCF3, a downstream transcription factor of the Wnt signaling pathway, can interact in the nucleus to synergistically enhance the expression of CPZ co-expressed genes and genes related to the Wnt signaling pathway, including WNT2/FZD2 and VIM, a marker gene for epithelial-mesenchymal transition. This process enhances the invasion and malignant metastasis of gastric cancer cells. In addition, studies targeting AEBP1 inhibitors may lead to new therapeutic strategies for gastric cancer patients, but more experiments are needed to validate this.

Despite relevant analyses and experimental validation being conducted, our study still has some limitations. The details of the interaction between AEBP1 and TCF3 are not yet clear in this research, and the specific domain and site of the interaction between the two should be further clarified through pull-down experiments based on protein truncations and mutations. Usually, one main TF works with distinct co-factors to transactivate or suppress different downstream. The major TF among AEBP1, TCF3, β-catenin and its co-regulators (such as histone deacetylase (HDAC) or homeodomain-interacting protein kinase 2 (HIPK2)) among the interaction of AEBP1 and TCF3 remains unclear. Moreover, whether AEBP1 or TCF3 is the necessary condition for VIM transcription under the activated Wnt signaling pathway needs further confirmation in the future. Last but not least, some statistical results may be biased due to sample size limitations and inter-individual differences. These inadequacies will be improved in our following studies.

## Conclusion

In short, the transcription of the Wnt signaling-related GC prognostic fingerprint gene CPZ and its co-expressed genes were promoted by the transcription factor AEBP1. A prognosis-associated gene expression program was found to be regulated by AEBP1 interaction with Wnt signaling downstream transcription factor TCF3, thus regulating the Wnt signaling, extracellular matrix, and EMT. Furthermore, AEBP1 knockdown and Wnt signaling inhibitor Quercetin synergistically inhibited the invasion and migration of gastric cancer cells, thereby restricting the peritoneal metastasis of GC. It appears that AEBP1 has potential effects on GC development and prognosis and may serve as a possible therapeutic target.

## Supplementary Materials

Figure S1 Expression of CPZ and its co-expressed genes in gastric cancer tissues. (A)CPZ expression was highly positively correlated with SERPINF1 expression in TCGASTAD.(B) SERPINF1 is specifically expressed in fibroblasts and tumor cells. (C)Expression heat map of CPZ co-expressed genes. *p < 0.05; **p < 0.01; ***p < 0.001.

Figure S2 Expression of AEBP1 in gastric cancer tissues and specific cell types. (A)AEBP1 and BGN expression levels are highly correlated. (B) BGN was specifically expressed in fibroblasts and tumor cells of gastric cancer tissues. (C) AEBP1 is specifically expressed in fibroblasts of normal gastric tissues (HPA database). (D) AEBP1 is highly expressed in gastric cancer tissues and low in adjacent tissues based on HPA database.

Figure S3 The impact of AEBP1 on the expression of its correlated genes.(A-D)Construction of HGC27 and MKN28 cell lines with AEBP1 shRNA stably knockdown. (E-F)AGS cell line with AEBP1 overexpression. EV: empty vector; OV: over-expression of AEBP1. *p < 0.05; **p < 0.01; ***p < 0.001.

## Data Availability

The datasets generated and/or analyzed during the current study are available from the corresponding author on reasonable request.

## Abbreviations

GC: Gastric cancer
LASSO: Least absolute shrinkage and selection operator
CPZ: Carboxypeptidase Z
CTHRC1: Collagen triple helix repeat containing-1
DKK1: Dickkopf-1
EGF: Epidermal growth factor
GPC3: Glypican proteoglycan 3
AEBP1: Adipocyte enhancer-binding protein 1
WNT2: Wnt family member 2
FZD2: Frizzled class receptor 2
VIM: Vimentin
PAPSS2: 3′-Phosphoadenosine 5′-phosphosulfate (PAPS) synthase 2
aKG: α-Ketoglutarate
CRC: Colorectal cancer
PTEN: Phosphatase and tensin homolog
EMT: Epithelial-mesenchymal transition
GSC: Glioblastoma
CRD: Cysteine-rich domain
ChIP: Chromatin immunoprecipitation
CREBBP: CREB binding protein
SMAD3: SMAD family member 3
CTNNB1: Catenin beta 1
HPA: Human Protein Atlas
KEGG: Kyoto Encyclopedia of Genes and Genomes
GO: Gene Ontology
GSEA: Gene set enrichment analysis
BioGRID: Biological General Repository for Interaction Datasets
STRING: Search Tool for Recurring Instances of Neighbouring Genes
PPI: Protein-protein interaction
SD: Standard deviation

## Supplementary Materials

The supplementary material is available online at https://doi.org/10.32604/or.2024.054366.
