# Supplementary material for: A novel Wnt/β-catenin signaling gene signature for progression and metastasis of gastric cancer
Source: Oncol Res. 2025 Apr 18;33(5):1199–215. doi: 10.32604/or.2024.054366 (PMC12035655; doi:10.32604/or.2024.054366)
Supplement: Figure S1 [file OncolRes-33-54366-s001.pdf]

Supplementary file 1

1. Figure S1-S3

2. Table S1-S5

Figure S1

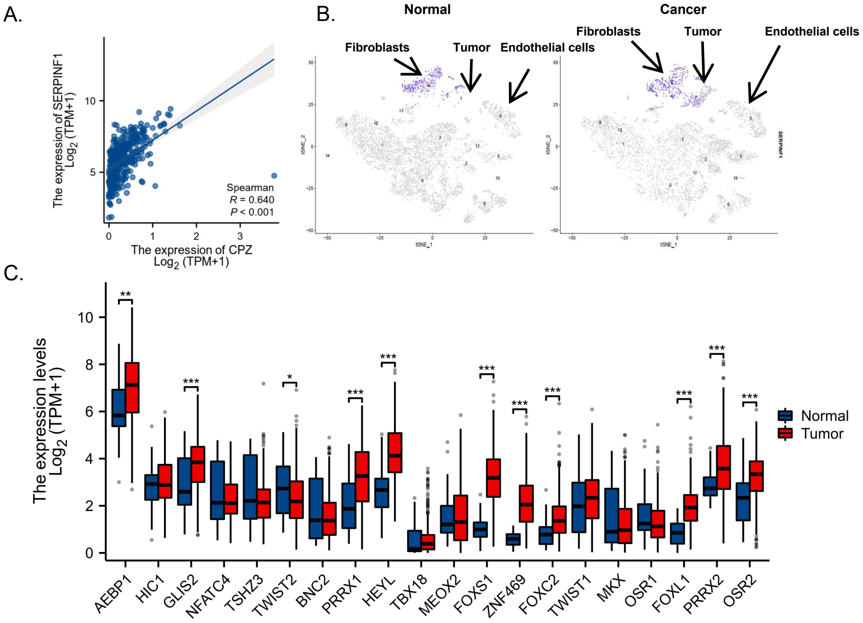

A.
The expression of SERPINF1
Log2 (TPM+1)
The expression of CPZ
Log2 (TPM+1)
Spearman
R = 0.640
P < 0.001
B.
Normal
Cancer
Fibroblasts
Tumor
Endothelial cells
tSNE_1
tSNE_2
C.
The expression levels
Log2 (TPM+1)
Normal
Tumor
AEBP1
HIC1
GLIS2
NFATC4
TSHZ3
TWIST2
BNC2
PRRX1
HEYL
TBX18
MEOX2
FOXS1
ZNF469
FOXC2
TWIST1
MKX
OSR1
FOXL1
PRRX2
OSR2

Figure S2.

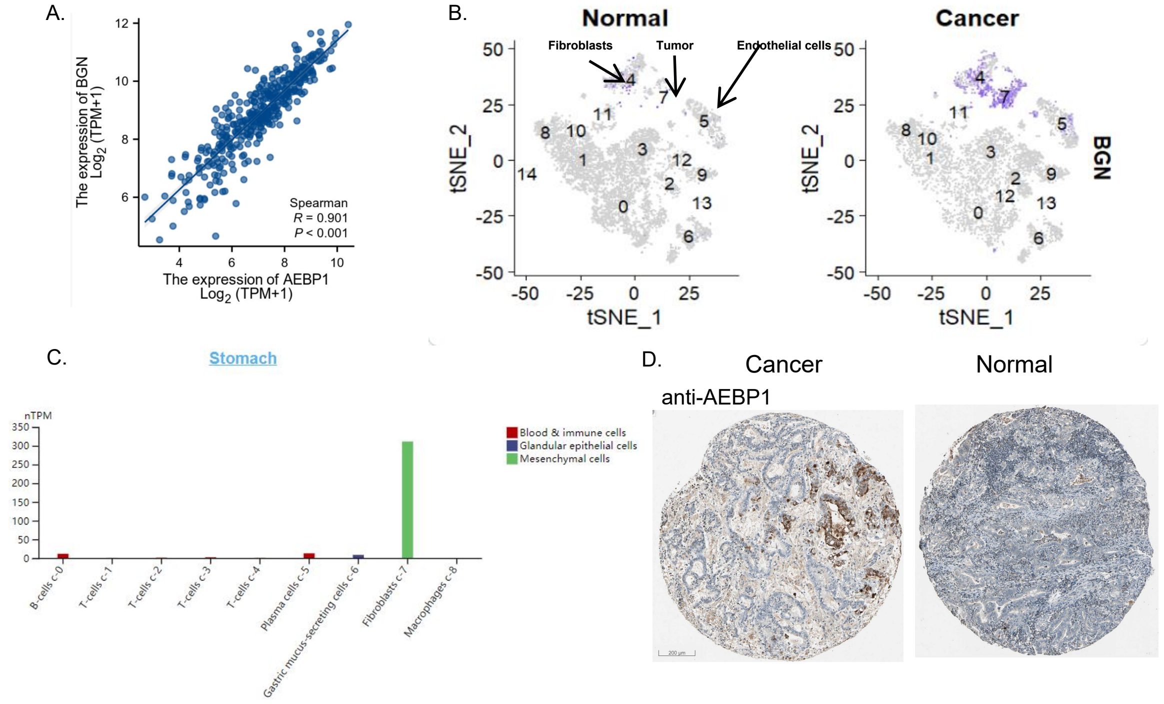
A.
The expression of BGN
Log2 (TPM+1)
The expression of AEBP1
Log2 (TPM+1)
Spearman
R = 0.901
P < 0.001
B.
Normal
Cancer
Fibroblasts
Tumor
Endothelial cells
tSNE_2
tSNE_1
BGN
C.
Stomach
nTPM
Blood & immune cells
Glandular epithelial cells
Mesenchymal cells
B-cells c-0
T-cells c-1
T-cells c-2
T-cells c-3
T-cells c-4
Plasma cells c-5
Gastric mucus-secreting cells c-6
Fibroblasts c-7
Macrophages c-8
D.
Cancer
Normal
anti-AEBP1

Figure S3.

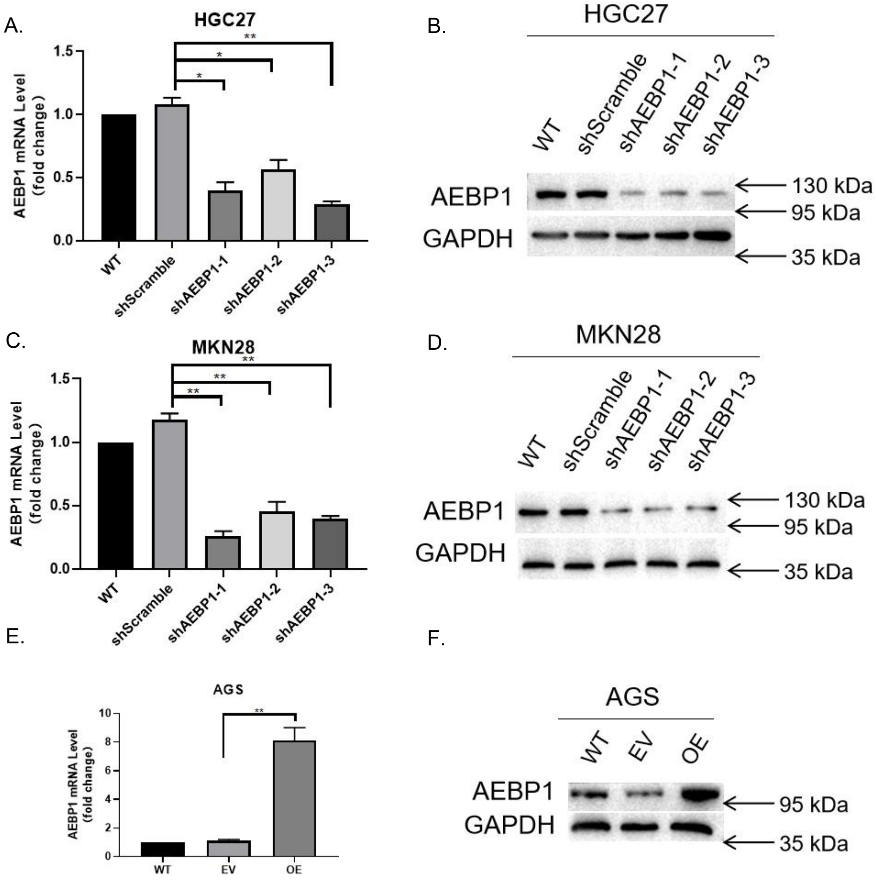

A.
HGC27
AEBP1 mRNA Level
(fold change)
1.5
1.0
0.5
0.0
WT
shScramble
shAEBP1-1
shAEBP1-2
shAEBP1-3
*
*
**
B.
HGC27
WT
shScramble
shAEBP1-1
shAEBP1-2
shAEBP1-3
AEBP1
GAPDH
130 kDa
95 kDa
35 kDa
HGC27
AEBP1 protein Level
(fold change)
1.5
1.0
0.5
0.0
WT
shScramble
shAEBP1-1
shAEBP1-2
shAEBP1-3
*
*
*
C.
MKN28
AEBP1 mRNA Level
(fold change)
1.5
1.0
0.5
0.0
WT
shScramble
shAEBP1-1
shAEBP1-2
shAEBP1-3
**
**
**
D.
MKN28
WT
shScramble
shAEBP1-1
shAEBP1-2
shAEBP1-3
AEBP1
GAPDH
130 kDa
95 kDa
35 kDa
MKN28
AEBP1 protein Level
(fold change)
1.5
1.0
0.5
0.0
WT
shScramble
shAEBP1-1
shAEBP1-2
shAEBP1-3
*
*
**
E.
AGS
AEBP1 mRNA Level
(fold change)
10
8
6
4
2
0
WT
EV
OE
**
F.
AGS
WT
EV
OE
AEBP1
GAPDH
95 kDa
35 kDa
AGS
AEBP1 protein Level
(fold change)
2.0
1.5
1.0
0.5
0.0
WT
EV
OE
*

Figure S1. Expression of *CPZ* and its co-expressed genes in gastric cancer tissues. (A) CPZ expression was highly positively correlated with SERPINF1 expression in TCGA-STAD. (B) SERPINF1 is specifically expressed in fibroblasts and tumor cells. (C) Expression heat map of CPZ co-expressed genes.

Figure S2. Expression of *AEBP1* in gastric cancer tissues and specific cell types. (A) *AEBP1* and *BGN* expression levels are highly correlated. (B) *BGN* was specifically expressed in fibroblasts and tumor cells of gastric cancer tissues. (C) *AEBP1* is specifically expressed in fibroblasts of normal gastric tissues (HPA database). (D) AEBP1 is highly expressed in gastric cancer tissues and low in adjacent tissues based on HPA database.

Figure S3. The impact of AEBP1 on the expression of its correlated genes.(A-D) Construction of HGC27 and MKN28 cell lines with *AEBP1* shRNA stably knockdown. (E-F) AGS cell line with *AEBP1* overexpression. EV: empty vector; OV: over-expression of *AEBP1*.

Table S1. Sequences of gene knockdown and scramble shRNAs used in this study.

Gene		Sequence(5'->3')	Target Sequence
shScramble for TCF3	Forward	CACCGGTCCAACCGATGCTTGAGTA CGAATACTCAAGCATCGGTTGGACC	GGTCCAACCGA TGCTTGAGTA
	Reverse	AAAAGGTCCAACCGATGCTTGAGTA TTCGTACTCAAGCATCGGTTGGACC	
shTCF3-1	Forward	CACCGGGATATTAACGAGGCCTTCC CGAAGGAAGGCCTCGTTAATATCCC	GGGATATTAAC GAGGCCTTCC
	Reverse	AAAAGGGATATTAACGAGGCCTTCC TTCGGGAAGGCCTCGTTAATATCCC	
shTCF3-2	Forward	CACCGGATATTAACGAGGCCTTCCG CGAACGGAAGGCCTCGTTAATATCC	GGATATTAACG AGGCCTTCCG
	Reverse	AAAAGGATATTAACGAGGCCTTCCG TTCGCGGAAGGCCTCGTTAATATCC	
shScramble for AEBP1	Forward	CACCGCGATGAAACGTGCGTCGTAA CGAATTACGACGCACGTTTCATCGC	GCGATGAAACG TGCGTCGTAA
	Reverse	AAAAGCGATGAAACGTGCGTCGTAA TTCGTTACGACGCACGTTTCATCGC	
shAEBP1-1	Forward	CACCGCCAGACATGGGTGATGTACA CGAATGTACATCACCCATGTCTGGC	GCCAGACATGG GTGATGTACA
	Reverse	AAAAGCCAGACATGGGTGATGTACA TTCGTGTACATCACCCATGTCTGGC	
shAEBP1-2	Forward	CACCGCTATGAGGAAATGACCTTTC CGAAGAAAGGTCATTTCCTCATAGC	GCTATGAGGAA ATGACCTTTC
	Reverse	AAAAGCTATGAGGAAATGACCTTTC TTCGGAAAGGTCATTTCCTCATAGC	
shAEBP1-3	Forward	CACCGCAATGTTGACTATGACATCG CGAACGATGTCATAGTCAACATTGC	GCAATGTTGAC TATGACATCG
	Reverse	AAAAGCAATGTTGACTATGACATCG TTCGCGATGTCATAGTCAACATTGC	

Table S2. Sequences of primers used for qRT-PCR in this study.

Gene		Sequence(5'->3')
AEBP1	Forward	AGACCACGCCATCTTCCG
	Reverse	CCTTGTTGTTCTCCCACTCG
FZD1	Forward	ATCTTCTTGTCCGGCTGTTACA
	Reverse	GTCCTCGGCGAACTTGTCATT
FZD8	Forward	ATCGGCTACAACTACACCTACA
	Reverse	GTACATGCTGCACAGGAAGAA
WNT2	Forward	CCGAGGTCAACTCTTCATGGT
	Reverse	CCTGGCACATTATCGCACAT
FZD2	Forward	GTGCCATCCTATCTCAGCTACA
	Reverse	CTGCATGTCTACCAAGTACGTG
FZD7	Forward	GTGCCAACGGCCTGATGTA
	Reverse	AGGTGAGAACGGTAAAGAGCG
CPZ	Forward	CTGCTGGTCATCGAGTTCTCC
	Reverse	TGCCACCTCATAGCCGTCA
FBLN2	Forward	ACTGTGGGTTCTTACCACTGT
	Reverse	CCACCTGGGAAAATTCTGACTT
EFEMP2	Forward	AAGAGCCCGACAGCTACAC
	Reverse	AGGGATGGTCAGACACTCGTT
TIMP2	Forward	AAGCGGTCAGTGAGAAGGAAG
	Reverse	GGGGCCGTGTAGATAAACTCTAT
COL8A1	Forward	GGGAGTGCTGCTTACCATTTC
	Reverse	AGCGGCTTGATCCCATAGTAG
FBN1	Forward	TTTAGCGTCCTACACGAGCC
	Reverse	CCATCCAGGGCAACAGTAAGC
β-Actin	Forward	CACCATTGGCAATGAGCGGTTC
	Reverse	AGGTCTTTGCGGATGTCCACGT

Table S3. Sequences of primers used for PCR in ChIP assay.

ChIP sequence		Sequence(5'->3')	Target Sequence
AEBP1 motif 1	Forward	ATTCTTCTCCCCGAGTCTTAA	ATTTC
	Reverse	CAGTTTAAAATCAACGGCAT	
AEBP1 motif 2	Forward	GAGGGCAAGGTTGAGCACCAG	GAAAT
	Reverse	TGCTTCCTGCAGGTCATCT	

Table S4. The explicit data of correlation coefficients and p-values.

	correlation coefficients (spearman)	P.adj
LTBP2	0.694293135	4.48535E-51
SRPX2	0.620930224	1.78509E-38
FBLN2	0.679122723	3.5856E-48
HMCN1	0.644018873	3.76723E-42
FBN1	0.653050441	1.03724E-43
THSD4	0.546840035	1.98129E-28
PODN	0.67298752	4.99917E-47
AEBP1	0.668863137	2.34875E-46
LAMA2	0.658674643	1.12388E-44
SRPX	0.557608443	9.45535E-30
COL8A1	0.657134546	1.9104E-44
COL16A1	0.584488899	3.07153E-33
ELN	0.631775546	3.79964E-40
EFEMP2	0.63249372	2.96816E-40
PHLDB1	0.619192977	3.35135E-38
LRP1	0.62168788	1.37521E-38
LTBP3	0.546751742	2.02766E-28
ADAMTS10	0.636316511	7.16892E-41
SH3PXD2B	0.615519794	1.14797E-37
MMP23B	0.570351521	2.36614E-31
SERPINF1	0.639997688	1.73383E-41
TIMP2	0.650822302	2.44798E-43

Table S5. The explicit data of correlation coefficients and p-values.

	correlation coefficients (spearman)	P.adj
AEBP1	0.668863137	2.34875E-46
HIC1	0.656396347	2.54641E-44
GLIS2	0.635495858	9.55451E-41
NFATC4	0.616540735	8.30943E-38
TSHZ3	0.612003175	3.78492E-37
TWIST2	0.607700705	1.64749E-36
BNC2	0.59566608	9.39238E-35
PRRX1	0.542980075	5.79071E-28
HEYL	0.542801783	6.06548E-28
TBX18	0.540114655	1.26163E-27
MEOX2	0.520991047	1.91871E-25
FOXS1	0.512652649	1.56459E-24
ZNF469	0.503016743	1.6402E-23
FOXC2	0.498259178	5.04679E-23
TWIST1	0.434481341	4.07387E-17
MKX	0.356216901	2.14762E-11
OSR1	0.355451789	2.40295E-11
FOXL1	0.342218695	1.58612E-10
PRRX2	0.287221893	1.56278E-07
OSR2	0.238675824	2.12673E-05
